# ‘Working Together to Meet in the Middle’. A Qualitative Descriptive Study of Power Dynamics in Lived Experience and Family Engagement in Mental Health and Substance Use Health Research

**DOI:** 10.1111/hex.70730

**Published:** 2026-06-29

**Authors:** Lisa D. Hawke, Joshua Dunphy, Natasha Yasmin Sheikhan, Wuraola Dada‐Philips, Shoshana Hauer, Charlotte Munro, Claudia Sendanyoye, Tanya Halsall, Gillian Strudwick, Yona Lunsky

**Affiliations:** ^1^ Centre for Addiction and Mental Health Toronto Canada; ^2^ University of Toronto Toronto Canada; ^3^ The Royal Ottawa Mental Health Centre Ottawa Canada

**Keywords:** lived experience, patient engagement, patient‐oriented research, power dynamics, qualitative research

## Abstract

**Background:**

Engaging people with lived experience (PWLE) and family members (/F) in mental health and/or substance use health research is an increasingly common practice and has many benefits. However, challenges are also encountered; these challenges include the experience of unequal power dynamics on research teams.

**Objective:**

This pragmatic qualitative descriptive study aimed to understand the experience of power dynamics in mental health/substance use health research engagement contexts, using an engagement framework.

**Method:**

Four focus group discussions were conducted with 18 PWLE/F aged 19–79 who had experience engaging in mental health and/or substance use research. A co‐designed semi‐structured interview guide was used. Discussions were audio recorded, transcribed, analysed using codebook thematic analysis and discussed with a Lived Experience Working Group.

**Results:**

Three primary themes were generated from the data: (1) Leadership, knowledge and expertise generate power. (2) Power dynamics should be acknowledged and balanced to improve research quality. (3) Power can be shared through authentic engagement practices.

**Conclusions:**

While knowledge and expertise generate power, researchers are not the only ones to hold knowledge and expertise, which necessitates the sharing of power. The mitigating strategies to help research teams address power are consistent with recommendations for conducting authentic engagement as a whole. Following best practices for PWLE engagement can help balance power dynamics on engaged research teams.

**Lived Experience Contribution:**

All aspects of this study were guided by a Lived Experience Working Group, from the identification of the research question to the interpretation and reporting of the results. People with lived experience and family members are co‐authors on this manuscript.

## 1 Introduction

People with lived experience of mental health and/or substance use challenges (PWLE) and family members (/F) can be engaged in research in roles such as advisors, collaborators and partners [1, 2]. They can be engaged throughout the research lifecycle, across research topics, including contributions to priority‐setting initiatives and preclinical research, through to implementation science and intervention scale [3, 4, 5]. A model of lived experience engagement proposes engagement on a spectrum that ranges from ‘informing’ patients through to ‘consulting’, ‘involving’, ‘collaborating with’ and ‘empowering’ [6].

PWLE/F bring substantial expertise regarding what works for them, including their health, service and research experiences and expectations. Common tasks in which PWLE/F are often engaged include setting priorities, discussing outcomes and outcome measures and reviewing participant‐facing documents [7]. They may also advise on study flows, recruitment and retention processes. Interpreting the data and contributing to both academic and lay knowledge translation activities are further common roles [7]. PWLE/F can contribute in many ways to improving the research endeavour. These include increased relevance of the research questions, positive impacts on PWLE/F and on researchers, improvements to the research processes and outcomes and more inclusive knowledge translation outputs [2].

Lived experience and family engagement in research, and specifically in mental health/substance use research, have emerged as a growing area of academic enquiry, framed as the science of engagement. Engagement can be a challenging process and has been found to have many barriers, as well as important facilitators [2]. Difficulties sometimes emerge in engagement due to PWLE/F feeling disconnected and researchers being ill‐equipped to conduct engagement, which is important given the risk of doing harm. Further barriers include power differentials, institutions failing to fully recognise the value of this work and teams engaging in a tokenistic way [2]. A recent scoping review of research evidence and implementation gaps identified a wide range of current gaps in the science of engagement [8]. Many of the gaps reflect the established barriers to engagement. These include relational factors such as how to build relationships and rapport, how to improve researchers' attitudes toward the engagement process, how to communicate clearly and, importantly, how to balance the power dynamics that can emerge in research engagement contexts.

The way power is distributed amongst team members, that is, power dynamics, has been examined in engagement contexts across health research. A qualitative study looked at power dynamics in community‐based participatory research and community‐academic partnership contexts in the United States [9]. In that study, racism was identified as a theme, as was the need for implicit bias training to address racism and improve communication. Authors also suggested ensuring structural competency and reflecting critically on the positionality of all involved. However, that study was conducted among a mix of patients and other community knowledge users, as well as researchers, and the PWLE/F were not specific to the mental health and substance use health research sector. Another study of the manners in which power can be shared among people engaged in health research, conducted across the United Kingdom and Australia, found that these were largely relational, with mechanisms such as active listening, effective facilitation, equal treatment and PWLE control over their work [10]. A collaborative group including patient partners developed a ‘power wheel’ to help research teams reflect on power imbalances, again across the health sciences [11].

In the mental health sector, power has been found to be a key factor that regulates when and how PWLE/F share the stories of their mental health journeys, which may also influence research engagement spaces [12]. While mental health and substance use health researchers have mentioned the potential impact of engagement on power differentials [13, 14], there is a lack of a rich understanding of how power plays out in lived experience and family engagement settings specific to mental health and substance use research. Lived experience and family engagement in mental health/substance use research have a number of characteristics that distinguish them from physical health research in terms of power dynamics. The inequities and oppression experienced by PWLE/F over the years, where recovery‐oriented, hope‐based care models have been rare due to the dominance of a deficit‐based approach, add to the power differential between PWLE/F and mental health professionals, including researchers [15, 16, 17]. This goes hand in hand with the historical and current stigma toward mental health and substance use health conditions [18, 19], which can also play out in the engagement process [20, 21]. All of this can lead to scepticism toward research, which can affect the research engagement process [2, 22] and potentially the sense of power differentials. It is possible that people living with mental health or substance use health concerns might be more vulnerable to the harms that can be caused by power imbalances and tokenistic engagement. Other issues that may be important across health, but heightened in the mental health and substance use health sector, include the importance of trauma‐informed practice, the role of social and structural determinants of health and challenges with regard to representativeness [23, 24], all of which can intersect with power. Given this diversity of factors, it is important to consider power dynamics specifically in the context of mental health and substance use health populations and research engagement settings.

Existing literature identifies power imbalances as a recurring barrier to engagement, but less is known about how PWLE/F understand the sources of power, when power is experienced as helpful versus harmful, and what practices help redistribute power in mental health and substance use health research. This study addresses that gap by examining power not only as a barrier to engagement, but as a relational feature of research collaborations that can be shared.

### 1.1 Objective

This qualitative descriptive study aimed to understand how PWLE/F engaged in mental health and substance use health research experience and navigated power dynamics on research teams, and what practices they identified as supporting more balanced power‐sharing. The topic emerged from the expressed priorities of PWLE/F and researchers in terms of advancing the science of lived experience and family engagement [25].

## 2 Methods

This was a qualitative focus group study supported by PWLE/F at all stages, from priority setting to knowledge translation. The study was guided by two overarching frameworks: (1) The Patient Engagement in Research conceptual framework, which guides the conduct and understanding of lived experience engagement [26], and (2) pragmatism as a paradigm, which is particularly well‐suited to research on engagement as a topic and to the practice of engagement in research [27]. Engagement is described using the GRIPP‐2 reporting guideline (Table 1) [28]. The qualitative approaches are described using the Standards for Reporting Qualitative Research reporting guidelines [29].

**Table 1:** Guidance for Reporting Involvement of Patients and the Public‐2 (GRIPP‐2) checklist.

Section and topic	Description
1: Aim	PWLE/F were involved in the project as a means of ensuring that all stages of the study were grounded in lived expertise.
2: Methods	A six‐member Lived Experience Working Group was recruited, although membership declined to three members by manuscript completion, due to pressures on individuals’ schedules. Virtual meetings were held with the team once or twice a month, which we scheduled flexibly. Meetings were facilitated with ice breakers and slide decks to support open discussion. Email communications occurred between meetings, and agenda summaries were circulated before the meetings. All PWLE/F received an hourly cash compensation for their time.
3: Study results	PWLE/F made many contributions to the project, for example (1) informing recruitment plans, (2) reviewing recruitment documents for inclusivity and accessibility, (3) revising the demographics form and the qualitative interview guide, (4) reviewed the qualitative themes and subthemes as well as the selected quotes, (5) discussing grants, conference presentations and papers related to the project, including co‐authorship of this manuscript, (6) completion of the GRIPP‐2 table.
4: Discussion and conclusions	Our engagement process helped the PWLE/F feel like trusted collaborators, thereby improving the study quality. The range of aspects of the study to which PWLE/F contributed ensured that the entire study was a relevant and meaningful project grounded in the lived expertise. The loss of Working Group members due to changing life circumstances was a challenge; two new members were added at the completion of these analyses. As is common with virtual engagement spaces, some technical challenges were encountered with the WebEx platform.
5: Reflections and critical perspective	PWLE/F reflected on their engagement in the project, and felt that they learned to be open and respectful of the voices of everyone in the room. The team addressed power dynamics as part of this study, building a safe space for the members. PWLE/F were invited to critique the project and suggest changes, freely. They were also given options for their involvement, as they were invited to choose contributions on the basis of their interests and skills. As a whole, the team felt that lived experience and family engagement generated a higher‐quality study through all the processes described above, and members experienced it positively.

### 2.1 Participants and Procedure

Eighteen participants were recruited through purposive sampling. Each participant participated in one of four virtual focus groups. Eligibility criteria included being aged 16 or over, residing in Canada and being a PWLE/F with experience engaging in research in roles such as advisors, collaborators, or co‐researchers within the mental health/substance use health sector. Four focus groups are considered a sufficient sample size, and saturation was achieved [30].

We recruited participants through multiple channels. Notably, the study was posted on the public research registry of the Centre for Addiction and Mental Health (CAMH). The study flyer was circulated to relevant organisations across Canada. Social media posts (X) were made for reposting by relevant bodies. This was supplemented by inviting Canada‐wide participants from two previous national projects on PWLE/F engagement led by the study lead at CAMH, who had consented to be contacted about future research. This recruitment strategy was selected to reach PWLE/F with direct experience in mental health and substance use health research engagement.

This study was conducted at CAMH, a specialised urban academic hospital in which PWLE/F engagement in research is common. Those interested in participating contacted the research staff by phone, text, or email. They were then screened for inclusion and underwent an informed consent process, including providing virtual signed informed consent. This was followed by the completion of a co‐designed demographic questionnaire on REDCap software [31]. Focus group discussions were then scheduled with consented participants as numbers allowed.

The focus groups were conducted following a co‐designed semi‐structured interview guide on the WebEx teleconferencing system on a secure server. They took about an hour and a half to complete (range 75–98 min. of interview discussion). Focus group facilitators were two research analysts (RAs) with experience in PWLE/F engagement in research. One participant preferred to attend in person and was provided with a laptop and a private space to do so to ensure accessibility; the remaining participants took part remotely. In addition, participants were assisted with WebEx navigation and troubleshooting any technical issues during the focus groups. The discussions were audio recorded and automatically transcribed by the WebEx system. An RA closely reviewed and corrected all automated transcripts. The focus group discussions were held between 19 September and 10 October 2024. All participants received a $50 gift card for participating. The study was approved by the CAMH Research Ethics Board.

### 2.2 Data Collection Instruments

A basic demographic form and semi‐structured interview guide were developed with input from the Lived Experience Working Group and pilot tested with the research staff team. They were built based on pragmatic considerations and Working Group interests in the topic rather than based on an externally developed theoretical framework, since lived experience team members are considered the direct experts in their research engagement experience and issues of importance to them. The demographic form collected basic information about the participants and their engagement backgrounds. The semi‐structured interview guide addressed a variety of related components of engagement, that is, relationships, power, rapport and communication, as experienced in PWLE/F engagement settings in the context of mental health and/or substance use research. The current manuscript presents the findings on power dynamics. Other results are reported elsewhere [32, 33].

### 2.3 Data Analysis

Codebook thematic analysis was used to analyse the transcripts [34], which is highly conducive to the iterative sharing process followed when people with lived experience are engaged in research. After three focus group discussions were completed and transcripts were cleaned, the lead researcher, the RA and an additional RA who had reviewed all transcripts met to discuss the content and build a codebook to represent the discussions. After programming the proposed codes into NVivo 12 [35], the transcripts were coded inductively into the codebook by an RA. The codebook was iteratively revised through ongoing discussion with the project lead, in relation to the data, by adding, renaming and collapsing codes as needed. Codes were then reviewed and organised into themes and subthemes to capture connected dimensions of power dynamics in engagement spaces. As the analysis was inductive, power frameworks were not used to generate the coding template, but informed later interpretation of the themes to situate participants' accounts within broader power literature.

The codebook, draft codes and preliminary themes were brought to the Lived Experience Working Group for discussion. Working group members provided feedback on the codes and themes, suggested refinements to theme structuring and wording and selected representative quotations. This process enhanced rigour and trustworthiness, in addition to improving the relevance of the analysis by ensuring it was informed by lived expertise.

### 2.4 Research Team Reflexivity

The research team brought varied lived, professional and academic experiences to this study. The lead data analyst (J.D.) is a mixed‐heritage man with an Honours Bachelor of Arts in Psychology. He is a person with lived experience, and this was his first experience analysing qualitative data generated by PWLE/F. The lead facilitator of the focus group discussions (W.D.‐P.) is a black woman with a Master's in Public Health. She is guided by PWLE/F, drawing on both her academic and lived expertise. The lead scientist (L.D.H.) is a white woman who works in a scientific position at CAMH. Her academic background is a PhD in psychology, and her current research platform focuses largely on lived experience and family engagement in mental health and substance use research. She has experienced both positive and problematic power dynamics in research engagement contexts and is committed to identifying means of optimisation. To support reflexivity, the team discussed emerging interpretations throughout the analytic process and brought preliminary themes to the Lived Experience Working Group for feedback.

## 3 Results

Participants were 18 PWLE/F with experience engaging in mental health and/or substance use research. They were 19–79 years old, including six young adults under the age of 30, seven adults aged 30–59 and two individuals aged 60 or over. The sample included eight women, six men and four transgender or non‐binary individuals. Ten were White, and the remaining participants were from a variety of ethnic/cultural backgrounds. Ten had been engaged as people with personal lived experience, two as family members, and six were engaged with both personal and family lived experience. The research they supported spanned the lifespan, including child and youth research (7 participants), adult research (15 participants) and geriatrics research (4 participants). The majority of participants were from Ontario, which is Canada's largest province and the location of the study team.

Across the focus groups, participants described power as both necessary and potentially harmful in research engagement contexts. Three themes were generated from the data, as illustrated in Table 2. The themes below show how power was generated through leadership and expertise, how it could be made visible and balanced and how authentic engagement practices functioned as mechanisms for redistributing power to PWLE/F.

**Table 2:** Themes and subthemes generated from the data.

Theme	Subtheme
1. Leadership, knowledge and expertise generate power.	1a. Researchers should show leadership.
	1b. Academic knowledge and expertise create power imbalances.
	1c. PWLE/F also have expertise and should therefore share the power.
2. Power dynamics should be acknowledged and balanced to improve research quality.	2a. Power dynamics need to be balanced.
	2b. To balance power, researchers should directly acknowledge and address power dynamics on the team.
	2c. Balanced power helps improve research quality.
3. Power can be shared through authentic engagement practices.	3a. Give PWLE/F opportunities to contribute meaningfully.
	3b. Ensure good communication practices.
	3c. Build mutually supportive research environments.
	3d. Respect and value PWLE/F as legitimate contributors and knowledge holders

### 3.1 Leadership, Knowledge and Expertise Generate Power

Participants described power as emerging from several sources, including leadership roles, academic expertise and the legitimacy granted to lived experience knowledge. Participants expressed that power is held based on who is in charge of a team, as power comes from the leadership role. They noted that knowledge and expertise create power imbalances. They did not frame all power differences as inherently problematic. Rather, participants distinguished between leadership that provided needed structure and direction and institutional authority that could become exclusionary when it positioned research knowledge as more legitimate than lived expertise. They further highlighted that PWLE/F hold expertise that should be recognised and that should come with power.

Participants described the importance of having researchers take the lead on a research project to ensure that the group functions effectively and purposefully. Without a leader, they felt that a research project would not make progress: ‘You need someone to enter the team to say “Ok, thank you all for your opinions. Here's where we're gonna go”*’* (Participant 1).

Either poor leadership or a lack of leadership can lead to power imbalances that will ultimately leave a research project directionless since ‘…you do need certain people on a team to have more knowledge on a topic…’ (Participant 2), which they felt the researchers can bring in their research leadership role. The necessity of a leader who is skilled in engagement is shown by a PWLE/F who recounted their experience on a team with a poorly performing leader:

[I] found out that this was his first experience in the research team, let alone been the leader of our research team (…). I noticed that in terms of power dynamics, a lot of these issues and problems can be resolved if you have an experienced leader on the team itself…

(Participant 1)

The leadership role comes with academic knowledge and expertise that can lead to power imbalances if not properly managed. One PWLE/F mentioned that they differentiate between the ‘hard goal structure of power’ and ‘…power when it comes to expertise and knowledge' (Participant 2). This interplay of expertise and power was illustrated by a participant who described that there were ‘knowledge barriers’ because during certain meetings they ‘would just be way out of my depth, totally lost at sea’ (Participant 3).

While complex terms and jargon drawn from academic expertise can make it difficult for PWLE/F to fully participate in a meeting, participants described that researchers should be able to estimate where the PWLE/F are at so they can avoid oversimplification, which can make PWLE/F feel as if the team is infantilising them. Participants described that PWLE/F notice when a research team takes steps to ‘dumb it down, because we've got somebody with lived experience on the group’ (Participant 1). It is important that researchers take note of the diverse knowledge and expertise of PWLE/F to ensure that communication is appropriate for their team.

As opposed to the academic expertise of researchers, PWLE/F also bring critical expertise through their lived experience, which provides them with a unique perspective on a research topic. This expertise merits power. If that lived expertise is valued, power can be balanced:

[We] definitely started gaining a bit more respect, I think, from the other members of the research team once they saw the potential advantages of having in‐depth, or, the depth of our unique perspectives.

(Participant 3)

Power can also be balanced when PWLE/F are given the opportunity to take on leadership roles, even if this occurs within a team that is formally led by a researcher. When PWLE/F ‘are the ones that are kind of leading’, and researchers are there to ‘to kind of monitor’, a participant expressed that they ‘kind of felt as if the power was shared’ (Participant 4) even though researchers were the ones making the final decisions in the experience of the participants.

Another way to share power is by giving PWLE/F tasks and responsibilities that allow them to use their lived expertise. For example, one participant shared that:

…those of us that felt comfortable doing it are putting together a separate paper of our own to publish on — sort of the — our experiences in the project as people with psychosis.

(Participant 3)

It is important to remember that lived expertise differs significantly from academic expertise. Researchers need to be skilled at cultivating a safe space that allows PWLE/F to share their unique lived experiences, which in mental health and substance use health research can be ‘sometimes very raw experiences’ (Participant 5). A lack of compassion from researchers may inhibit PWLE/F from sharing their lived experiences:

And I'm there as a lived experience partner and that's where it shuts down some of my need to share, because I can sense that there's maybe not authentic, connection being made there that is more of an agenda.

(Participant 5)

If researchers truly value the expertise of PWLE/F as being of equivalent value, then power can be more evenly shared.

### 3.2 Power Dynamics Should Be Acknowledged and Balanced to Improve Research Quality

Participants framed acknowledging power as a condition for meaningful engagement. Rather than assuming that engagement research spaces were automatically equal, they described the reasons why power should be balanced, how it can be balanced through acknowledgement, and the fact that balanced power improves the quality of research. Naming power helped participants assess whether their involvement was likely to be influential versus symbolic.

Participants noted the need to balance out the power that comes with leadership so that everyone is included. For example, one participant said, ‘…it is a little beneficial to have a little bit of power dynamics within a research team…,’ however, it's important that, ‘…everyone's opinions should still be taken into regards…’ (Participant 2). PWLE/F pick up on poorly balanced power, which may be a common occurrence:

It's always been like the lived experience person is sort of like a bit of a lower rank person, just because you're the invited guest as opposed to the one who's, e.g., co‐facilitating, which would be like, I think equal power kind of thing, which I've never seen.

(Participant 5)

If power is unbalanced, then a team risks losing out on engagement and ideas from PWLE/F, as expressed by someone who said, ‘I've worked in a team where the team lead kind of made it very obvious that there was that hierarchy’ (Participant 6), which led them to disengage from the project. They went on to reflect that a while, ‘…everyone knows there's a hierarchy,’ if the researchers fail to properly address or balance this power differential, the research team will, ‘…lose respect or good ideas that might be helpful’ (Participant 6).

Researchers should take explicit action to ensure that existing power dynamics are addressed and the power is distributed among the team. By reflecting on power and the imbalances in place, researchers can be prepared to address them directly with the team to improve engagement processes. For example, one participant noted that openly stating that researchers are in a position of power without tactfully discussing it will ‘…naturally lead to greater power dynamics…’ (Participant 4). In contrast, another participant described a positive example of power dynamics being addressed by the researcher: ‘it was actually one of the researchers who checked themselves, so I guess a high level of insight among in the research team and sort of higher ups ’ (Participant 3).

In a similar vein, one participant noted that when PWLE/F and researchers were ‘…working together to meet in the middle’ (Participant 7), power felt more evenly distributed since those in leadership positions approached conflict resolution cooperatively. Participants also noted that by ‘knowing up front who has the power in the room…,’ PWLE/F can learn about expectations and whether or not their participation is ‘performance art or whether it can really make a difference’ (Participant 8). This participant provided a clear example of this understanding of the power dynamic:

[P]eople will see those in lab coats holding more authority and power, and I think this needs to be identified and just brought to the forefront so people feel like this is an open forum…

(Participant 8)

Being open about the power that a researcher holds makes PWLE/F more comfortable to speak out, which can help address the times when PWLE/F ‘may feel a little inferior’ (Participant 1).

Participants felt that balancing power by valuing the expertise of PWLE/F improves research quality since ‘who better to talk about something than someone who's been through it?’ (Participant 9). Participants expressed a variety of ways that balanced power improved their research project. For example, one participant noted that they were able to get the researchers to work with ‘more compassion,’ and they were able to convince the team to ‘collect a new type of information on people’ (Participant 3).

When power is addressed and balanced with PWLE/F involved as valued collaborators, research projects tend to be ‘more of a story,’ rather than simply ‘…these are the points and these are the data, this is the data set and that's it’ (Participant 10). Participants felt that when power dynamics are balanced in this way, the research becomes more humanistic, as scientists may be biased towards a more scientific or mechanistic lens. One participant succinctly said that ‘well, it's valuing what people have to say, really valuing it because it makes your research better’ (Participant 11).

### 3.3 Power Can Be Shared Through Authentic Engagement Practices

Participants described authentic engagement practices as practical mechanisms for sharing and redistributing power. Clear communication, meaningful roles, supportive environments, recognition and respect were ways through which PWLE/F gained voice, influence and legitimacy within research teams.

Power dynamics can be balanced by using good communication as the foundation of authentic engagement. Communication needs to be shared, authentic and collaborative to ensure that PWLE/F feel engaged and appreciated, which in turn helps to balance power imbalances. Good communication can be established by expressing genuine interest in PWLE/F and their unique experiences. A common sentiment among PWLE/F was that they could sense when researchers were uninterested in having PWLE/F as part of their team:

…the facilitator should want the people to participate in her heart and brain, I guess, because that can be read. If she's just reading a script, you know that as well, it's cold. You know, we can sort of sense fakeness.

(Participant 11)

PWLE/F can sense when someone is being inauthentic due to poorly balanced, disengaged and robotic communication styles. Those who are given more time to take the floor may have more power:

…if we measure the power dynamics by how much time you have, let's say on the floor or you're, you're taking time, that could be a measure, I think, of power.

(Participant 5)

When adopting ‘a collaborative style and open communication style, [power is] more evenly distributed…’ (Participant 5). Communication styles that are more assertive also tend to overpower ‘people who kind of will give their input more softly…’ (Participant 2).

In addition to communication, providing PWLE/F with opportunities to meaningfully contribute to a research project was considered to help balance power dynamics, since engagement in research tasks can be indicative of how much power someone holds. One participant described that as their responsibilities increased, so did their ‘…quote‐unquote power within the research…’ (Participant 12). There are many ways in which PWLE/F can contribute meaningfully to projects, depending on their skills and interests. Participants felt that it was important for the researchers to provide ‘creative ways’ (Participant 3) to contribute in a manner that played to their strengths. This helps ensure PWLE/F are meaningfully included, balancing power regardless of their prior experience, skill set, or professional expertise. Participants also noted that being formally credited for their contributions was a meaningful way of addressing power imbalances in their lab. For instance, one participant said that,

When it comes to power, I feel like I've had very pleasant experiences with feeling like equal among everyone within the team, and that's also done through like being able to be labelled a co‐researcher or being able to have the opportunity to contribute to like a paper, and have my name attached to it.

(Participant 13)

Another participant noted that their greater involvement meant their ‘…role on the group was a full member of the group,’ rather than feeling as if they were ‘just there to make up the numbers’ (Participant 1). Another way that participants felt that power could be shared was by cultivating a supportive research environment as an authentic engagement practice. This could be done by ‘…providing training on, like, diversity, equity and inclusion,’ which can ‘raise awareness about power dynamics…’ (Participant 2). Participants felt that educating a research team on diversity helps to create an understanding environment, which in turn educates PWLE/F and researchers on power dynamics. By gaining a greater understanding and appreciation of the unique characteristics of people involved in research, research teams can become more aware of how these unique characteristics might contribute to the research setting's power dynamics. Participants also indicated that power was shared by social location beyond formal research roles. For example, one participant noted that ‘socioeconomic status’ and factors beyond a person's ‘actual job role’ (Participant 7) could shape power and authority within a team.

Mutually supportive research environments can improve the power structure in place by creating a ‘very comfortable environment’ (Participant 2) where people are not ‘strangers to each other’ and the team ‘doesn't quite feel like a work environment so you don't feel stressed in that sense’ (Participant 2). This kind of supportive environment can help PWLE/F address potential power imbalances within their team. When PWLE/F feel supported in their team, they may feel more comfortable addressing power imbalances. Thus, when a research team creates a safe place for conflict resolution and cooperation, PWLE/F and researchers are better equipped to address power imbalances.

Participants felt that valuing and respecting PWLE/F, as a core component of authentic engagement, further balances power dynamics. When PWLE/F are treated with respect, they feel more appreciated and equal in power to the research team: ‘…you're treated equally and with respect. And, so they make you feel you're part of the group. You're not an outsider looking in per se…’ (Participant 9).

Another participant described how power was balanced on their team through value and respect of PWLE/F,

…the group I was with, they did genuinely see value and having someone like me on the group, not just to contribute in terms of putting the structure of the research together, also able to go to the treatment center and to speak to patients…

(Participant 1)

In contrast with sharing power by showing genuine respect for PWLE/F, disrespect can exacerbate power imbalances by lessening the involvement of PWLE/F and can lead to ‘disengagement and just removing yourself from an entire product’ (Participant 5).

## 4 Discussion

This qualitative descriptive study examined power dynamics in the engagement of PWLE/F in mental health and substance use health research. Three primary themes were generated from the data: (1) Leadership, knowledge and expertise generate power. (2) Power dynamics should be acknowledged and balanced to improve research quality. (3) Power can be shared through authentic engagement practices. While knowledge and expertise generate power, researchers are not the only ones to hold knowledge and expertise, necessitating the acknowledgement of power structures and the active sharing of power, which can be challenging within inherently hierarchical academic research settings. The mitigating strategies to help research teams address and balance power were considered to be consistent with recommendations for conducting authentic engagement as a whole.

Previous studies have pointed to challenges associated with unequal power dynamics [2, 9, 12]. This study is unique in its finding that power is not always perceived to be fundamentally a problem, as there is power inherent to the leadership required to conduct the research. Indeed, a research engagement setting without a sense of leadership might lead to unclear project plans, a lack of identified tasks for advisors and role confusion, all of which are barriers to effective engagement [2]. However, that leadership does not have to come from the researchers, but can also be shared with people with lived experience. Within the context of strong leadership, efforts are still required to balance power in a way that gives everyone the opportunity to be respected for the expertise that they bring to the project and to contribute meaningfully. Given a complex history of exclusion, marginalisation, stigma and coercion in mental health and substance use health research and care [15, 36], it may be particularly important to attend to power in this sector of research as a matter of epistemic justice and to address past harms.

The workplace management literature describes a range of types of power in the personal and organisational spheres, which have not been applied to lived experience and family engagement contexts in the health research literature but which may help understand the complexities of power in research engagement. One model describes organisational power as including an individual's legitimacy due to a higher role held in the organisation, the ability to support subordinates by providing rewards and coercive power to apply punishments [37]. Based on this model, personal power is divided into expert power based on their expertise and referent power based on admiration and respect [37]. In the current study, PWLE/F acknowledged that researchers do have organisational power in that they hold a more senior position with the institution and are in a position to give rewards (i.e., compensation, recognition). The results suggest that personal power, both in the form of expert power and referent power, should both be shared by researchers and PWLE/F, where lived and learned experience are equally valued and everyone is respected for the expertise they bring to the team. Researchers might consider extending beyond the engagement literature and into the workplace management literature to help to categorise and understand power dynamics and how they apply in PWLE/F research engagement settings.

Applying strong engagement practices was seen as a primary mitigating strategy to reduce power imbalances on research teams. Notably, strong engagement practices included attention to communication, authentic respect, meaningful opportunities and supportive environments. A number of pragmatic, PWLE/F‐focused recommendations exist to guide research teams in best practices in engagement, in the form of scientific manuscripts and online tools offered by funding bodies and various interest groups [26, 38, 39, 40, 41]. The various sources of guidance converge on the notions raised by our participants, focusing on meaningful contributions, clear communication of roles and expectations, respect for their contributions, providing support and acknowledging contributions. Authentic and meaningful engagement practices as a whole, then, may be so important in large part because they serve to balance the power dynamics. Researchers are encouraged to apply the best practices for PWLE/F engagement in their work with a goal of improving all aspects of engagement, including issues of power [41].

It is important to consider the broader services side of the mental health and substance use healthcare system. In that system, service providers inherently hold power over patients by virtue of their training, authority and ability to offer or withhold care [42]. Patient‐oriented care approaches centre patient autonomy and choice as reparative mechanisms [43, 44]. By giving patients autonomy and choice, it is considered that trust in and the legitimacy of healthcare may be augmented [43, 44]. This reasoning can be extended to research engagement. Indeed, a degree of lived experience choice and autonomy—in how they engage and in what they contribute—appears to be essential to balancing power dynamics in healthcare research engagement settings. Balancing power in engagement settings could therefore augment trust in mental health and substance use health research, which may be particularly important in certain current healthcare climates.

Mental health and substance use health have a number of particularities that justify work specific to this sector. Extensive injustices and oppression make mental health and substance use research a priority area for PWLE/F engagement [15] and for examining power dynamics. Stigma toward mental illness and substance use is common in healthcare and impacts engagement [21, 45, 46]. Trauma and representativeness must be attended to given the intersections between mental health, substance use, trauma and social and structural determinants of mental health [47]. However, power dynamics have also emerged as a topic of interest in patient engagement in the physical health sphere [48]. It is possible that some of the current findings may apply outside of mental health and substance use. For example, the application of best practices in engagement as a means of balancing power dynamics may find application in the science of engagement as a whole. This might be considered by researchers and PWLE/F across the health research sector, including physical health, maybe especially so in research on sensitive or stigmatised health issues.

### 4.1 Strengths, Limitations and Implications for Future Research

This study has strengths and limitations to consider when interpreting the findings. It was a lived experience‐engaged study; PWLE/F supported all stages, from the early priority setting stage to reporting, strengthening the relevance and trustworthiness of the conceptual underpinning of the study, the analysis and the interpretation and reporting. Participants were also diverse in age and represented research across the lifespan.

Several limitations should be noted. While six participants had family lived experience, only two participants identified as engaging strictly on the basis of family lived experience, limiting the findings specific to family members who are not also engaging from the perspective of their personal lived experience. While a degree of demographic diversity was achieved, including gender diversity, this study was not designed to examine how power dynamics may differ across social locations or intersecting identities. As such, the findings may not fully capture how racism, colonialism, ableism, transphobia, housing precarity and other structural inequities shape experiences of power in engagement spaces. This is an important gap for future research as power dynamics in engagement are unlikely to be experienced uniformly across groups.

Recruitment also drew in part from prior Canada‐wide projects led by the same institution, which may have introduced bias through repeated voices. The study was also conducted within an institutional context where engagement is common and positively regarded, and by a team committed to improving engagement practices. This context may have shaped who chose to participate, how participants may have framed their experiences, and how the research team interpreted accounts of power dynamics.

Note that this study examined mental health and substance use health together, given the high rates of comorbidity and the common practice of researching and engaging with both topics together; however, there could be some differences in power dynamics in mental health versus substance use health settings, which is an area for future research. Future research should also examine: (1) researchers' perspectives on sharing power in lived experience and family engagement contexts; (2) how power dynamics and power sharing with PWLE/F exist on the backdrop of the power structures built into research institutions and the discipline as a whole; (3) power sharing within structurally marginalised groups, such as Indigenous, 2SLGBTQI+, ethnically diverse and homeless or precariously housed participants, those with disabilities and outside of the Canadian context; and (4) the relationship between power sharing and trust in the resulting research.

## 5 Conclusions

Researchers hold power in the lived experience and family engagement process by virtue of their leadership role and research knowledge. However, PWLE/F also bring expertise to the team, and power must therefore be shared. Power can be balanced by following best practices for authentic lived experience and family engagement in mental health and substance use health contexts. By following best practices and communicating clearly with PWLE/F in supportive research environments, researchers can ensure that PWLE/F are authentically respected and have meaningful opportunities to contribute to the research at hand, thereby generating the positive impacts expected of PWLE/F engagement activities.

## Author Contributions

**Lisa D. Hawke:** conceptualisation, investigation, funding acquisition, writing – original draft, methodology, validation, formal analysis, project administration, supervision, resources, data curation. **Joshua Dunphy:** writing – original draft, writing – review and editing, formal analysis, conceptualisation. **Natasha Yasmin Sheikhan:** conceptualisation, methodology, writing – review and editing. **Wuraola Dada‐Philips:** conceptualisation, writing – review and editing, methodology, investigation, formal analysis. **Shoshana Hauer:** conceptualisation, methodology, writing – review and editing, formal analysis. **Charlotte Munro:** conceptualisation, methodology, writing – review and editing, formal analysis. **Claudia Sendanyoye:** conceptualisation, writing – review and editing, methodology, formal analysis. **Tanya Halsall:** conceptualisation, writing – review and editing, methodology. **Gillian Strudwick:** conceptualisation, methodology, writing – review and editing. **Yona Lunsky:** conceptualisation, methodology, writing – review and editing.

## Conflicts of Interest

The authors declare no conflicts of interest.

## Data Availability

The data underlying this project are subject to the Research Ethics Board of the Centre for Addiction and Mental Health. Data may be shared upon reasonable request to the author and with Research Ethics Board approval.
